# Lésion papulo-squameuse chronique: penser au papillome

**DOI:** 10.11604/pamj.2018.29.133.11169

**Published:** 2018-02-24

**Authors:** Marie Ida Rahantamalala, Ny Ony Andrianandrasana

**Affiliations:** 1 Service Médecine Interne, Hôpital Ambohidratrimo Anosiala, CHU Antananarivo, Madagascar; 2 Service Oncologie et Radiothérapie, Hôpital Joseph Ravoahangy Andrianavalona, CHU Antananarivo, Madagascar

**Keywords:** Papillome, épithélium malpighien, coude, Papilloma, malpighian epithelium, elbow

## Abstract

Papilloma is a benign tumor of the malpighian layer, usualy affecting the skin. Thickened epithelium doesn’t show cytological or architectural abnormalities; the epithelial proliferation is still clearly separated from the dermis by the basal membrane, that is to say absence of tumor infiltration. We here report the case of a 15-year-old boy presenting with oozing plague in his left elbow that had evolved over the previous two months. The patient had no particular previous history. Physical examination showed good general condition and a papular, squamous, itchy, oozing lesion measuring 4 cm along its longer axis. The remainder of the physical examination was normal. He was treated with topical emollient, antimycotics and quinolone antibiotics. Drying of the purulent oozing was observed, but no improvement in the general appearance of the lesion. Laboratory tests, including glucose tolerance test, were normal. A biopsy of the lesion was performed. Anatomo-pathological examination of skin biopsy specimen showed no abnormal cells, rather an elongation of the epidermal ridges as well as of the connective papillae, extensive keratinization of the surface and thickening of the malpighian layer. The diagnosis of papilloma was retained. Surgical excision of the tumor resulted in complete healing.

## Image en médecine

Le papillome est une tumeur bénigne du revêtement malpighien, et a comme localisation habituelle la peau. Il n’y a pas d’anomalies cytologiques ou architecturales au niveau de l’épithélium épaissi ; la prolifération épithéliale est toujours nettement séparée du derme par la membrane basale c'est-à-dire absence de toute infiltration. Il s’agissait d’un adolescent de 15 ans, venu en consultation pour une plaie suintante au niveau du coude gauche, évoluant depuis deux mois auparavant. Il n’avait pas d’antécédent particulier. A l’examen physique, il était en bon état général, et présentait une lésion papuleuse, squameuse, prurigineuse, de 4cm de grand axe, suintante. Le reste de l’examen était normal. Il a été traité par des traitements locaux par émollient, antimycosiques et antibiotiques à base de quinolone. On notait seulement un assèchement du suintement purulent, mais aucune amélioration par rapport à l’aspect général de la lésion. Les bilans biologiques notamment l’hyperglycémie provoquée étaient normaux. Une biopsie de la lésion avait été réalisée. L’examen anatomo-pathologique du fragment biopsique épidermique ne montrait pas de cellules anormales, plutôt un allongement des crêtes épidermiques et conjointement des papilles conjonctives, une importante kératinisation de surface et un épaississement du revêtement malpighien. Le diagnostic de papillome était retenu. L’exérèse chirurgicale de la tumeur permettait une guérison complète.

**Figure 1:**
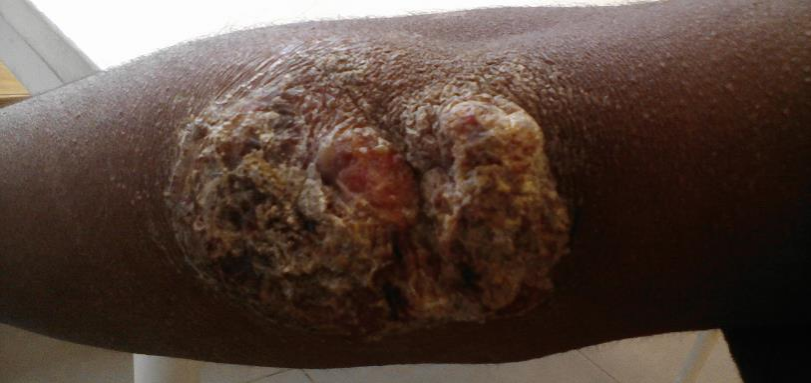
papillome du coude gauche

